# Chronic grouped social restriction triggers long-lasting immune system adaptations

**DOI:** 10.18632/oncotarget.16856

**Published:** 2017-04-05

**Authors:** Rui Tian, Gonglin Hou, Liuwei Song, Jianming Zhang, Ti-Fei Yuan

**Affiliations:** ^1^ Department of Psychology, Zhejiang Sci-Tech University, Hangzhou, China; ^2^ State Key Laboratory of Molecular Vaccinology and Molecular Diagnostics and Center for Molecular Imaging and Translational Medicine, School of Public Health, Xiamen University, Xiamen, China; ^3^ National Institute of Diagnostics and Vaccine Development in Infectious Diseases, Xiamen University, Xiamen, China; ^4^ School of Psychology, Nanjing Normal University, Nanjing, China; ^5^ Shanghai Key Laboratory of Psychotic Disorders, Shanghai Mental Health Center, Shanghai Jiao Tong University School of Medicine, Shanghai Jiao Tong University, Shanghai, China; ^6^ State Key Laboratory of Brain and Cognitive Sciences, The University of Hong Kong, Hong Kong

**Keywords:** chronic stress, cytokines, chemokines, social restriction, human

## Abstract

Chronic stress triggers rigorous psychological and physiological changes, including immunological system adaptations. However, the effects of long-term social restriction on human immune system have not been investigated. The present study is to investigate the effect of chronic stress on immune changes in human blood, with the stress stimuli controlled.10 male volunteers were group isolated from the modern society in a 50-meter-square room for 150 days, with enriched nutrition and good living conditions provided. Serum examination of immune system markers demonstrated numerous changes in different aspects of the immune functions. The changes were observed as early as 30 days and could last for another 150 days after the termination of the restriction period (300 days’ time point). The results strongly argued for the adaptation of immunological system under chronic social restriction stress in adult human, preceding a clear change in psychological conditions. The changes of these immune system factors could as well act as the serum biomarkers in clinical early-diagnosis of stress-related disorders.

## INTRODUCTION

Chronic stress leads to dynamic changes in psychological conditions and immunological systems [1, 2]. The common chronic psychosocial stress stimulus includes job stress (burnout), low socioeconomic status (SES), childhood maltreatment/adversities, caregiver stress (e.g. nurses), and aged loneliness [3]. These types of stress stimuli modify the body inflammation signaling, especially the circulated cytokines [4–6]. The cytokines can be divided into different groups including interleukins, growth factors, chemokines, tumor necrosis factors, colony-stimulating factor and Interferons. All of them plays an important role in immune responses by aiding cell to cell communication and stimulate the movement of cells towards sites of inflammation, infection and trauma. However, the potential effects of social restriction on human immune system have not been investigated.

In present study, we aimed to investigate the effects of chronic social restriction stress (150 days) on the immune system function (cytokine levels and immune cells) of ten healthy volunteers. The results proved the inflammatory sequels of social restriction, and provided certain potential biomarkers for stress-resulted psychological diseases. Interestingly, the immune changes might precede a clear psychological effect, arguing for a potentially causing role of immune system dysfunction in stress-relevant brain disorders.

## RESULTS

### Chronic social restriction leads to cortisol increase

Surprisingly, we found that the social restriction leads to only mild changes in psychological evaluation scores when looked at the average values from the 10 volunteers (Supplementary Figure 1A). Specifically, the restriction leads to weakly increased hostility against each other in the social restriction period, especially in the first 90 days; while the self-reported emotion status exhibited slightly increased anxiety in the first 90 days, decreased a bit at 125 days and raised up again at the end of the stress period.

**Figure 1:**
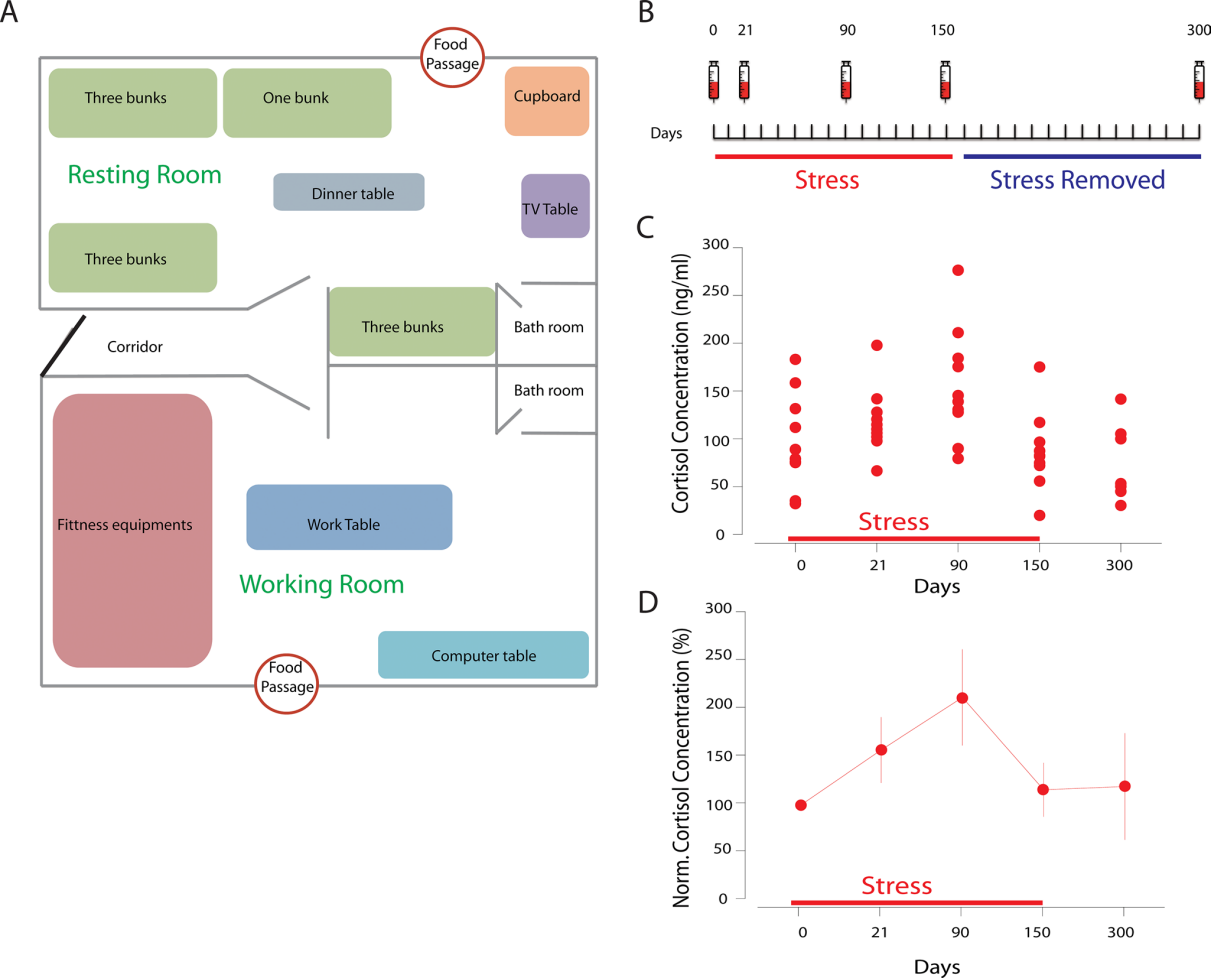
(**A**) the design of the room. The resting room is 670 × 350 × 220 cm, about 24 square meters, equipped with beds, TV and dinner table; the working room is 670 × 385 × 220 cm, about 26 square meters, equipped with fitness equipment and computers. The humidity was 35-60%; the temperature was 23.3-26.0 degree. All subjects received sunshine at given period during the restriction. The activity time and sleep time were both more than 8 hours. (**B**) the design of the blood withdrawal schedule in the morning of the day points; (**C**) stress triggered serum cortisol increase during the experimental period, showing individual results. (**D**) the serum cortisol increase normalized to day 0.

In order to explore potential changes of the immune system of the subjects, we decided to directly measure the circulating cytokines within the serum. We collected peripheral blood samples on the day before the initial test (day 0) for control, at the 21 days (day 21), 90 (day 90), 150 days after the start of the test (day 150), and at the 150 days after the restriction was stopped (day 300) (Figure 1B). We found that the basal level of cortisol is normal to other healthy subjects, validating the use of day 0 as the control.

During the stress period, we found that the social restriction effectively and rapidly triggered serum cortisol increase as early as 21 days, which lasted during the whole isolation period (Figure 1C, Supplementary Figure 2), in a line with the effect of social restriction stress. Yet at 150 days the cortisol level was lower than that at 90 days, suggesting for the adaption to the environment, familiarity with each other and/or the expectation to the free of the stress (end of the experiment), and it may also because of the hypothalamic-pituitary-adrenocortical (HPA) axis fatigue under long-term stress. In addition, the social restriction stress induces increases of other stress-related hormones as expected, such as triiodothyronine (T3) and testosterone (Supplementary Figures 3, 4).

### Chronic social restriction results in long-lasting cytokine changes

**Figure 2:**
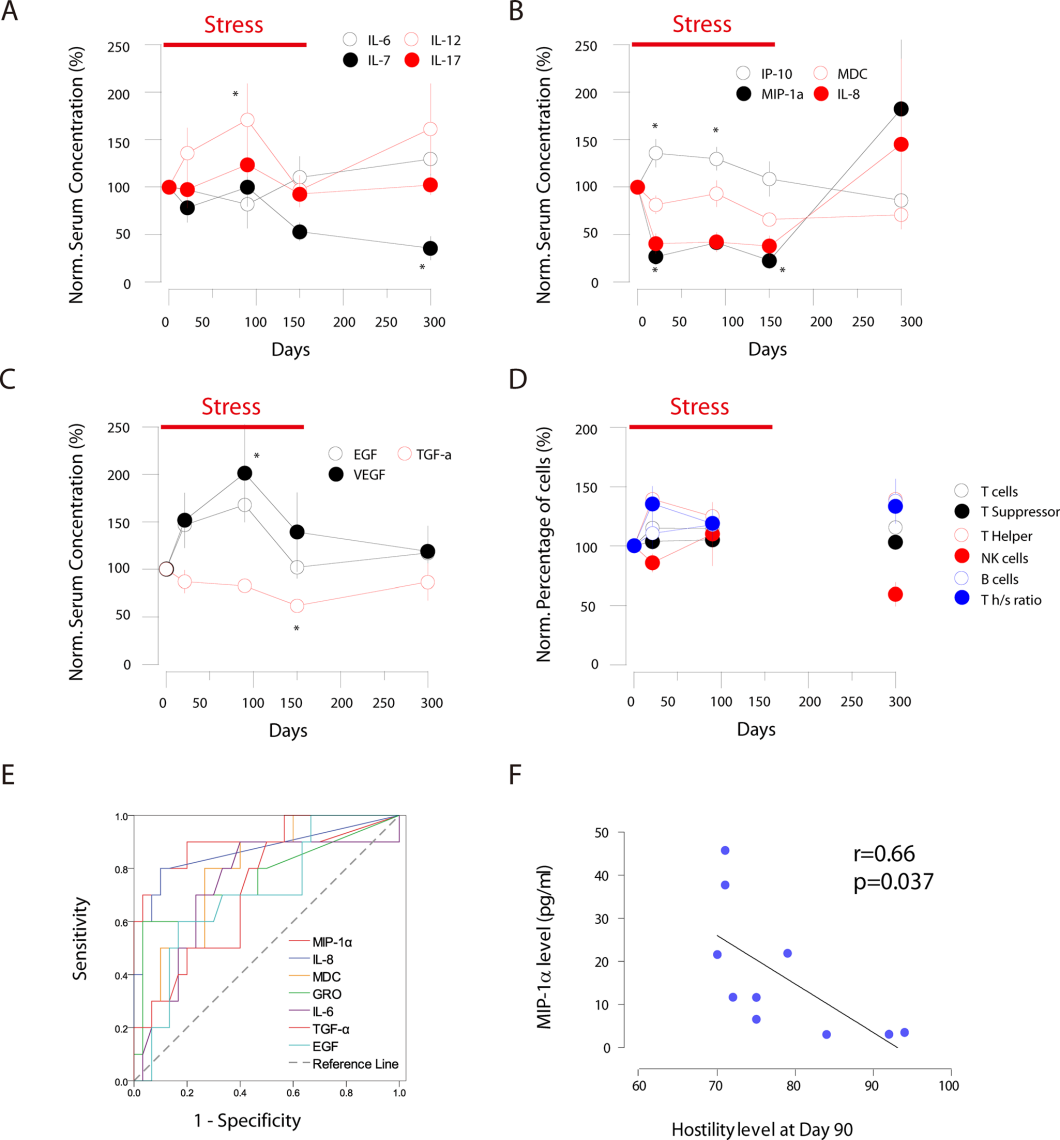
2ABC: changes of different cytokines families during the stress period (Normalized to day 0) (**A**) interleukin family cytokines; (**B**) chemokine family cytokines; (**C**) growth factor family cytokines; (**D**) changes of different groups of lymph cells (Normalized to day 0); (**E**) The ROC curve analysis of cytokine as the biomarker to stress-related disorders; (**F**) MIP-1α serum concentrations in correlated to hostility level across different individuals at day 90, suggesting for the varied vulnerability to stress. * Indicates that the *P* < 0.05 in compared to baseline level.

We further examined the levels of different groups of cytokines (a total of 19) in the serum from the 10 subjects with MILLIPLEX MAP Human Cytokine/Chemokine Magnetic Bead Panel - Premixed 39 Plex and Luminex 200 Total System. Within the interleukin family cytokines, IL-7 exhibited long-lasting decrease which did not return to the baseline even at 300 days after the start of the restriction stress; while IL-12 exhibited periodic increase in the serum during the study. IL-6 and IL-17 showed mild alterations (Figure 2A, Supplementary Figure 5). Among chemokine family members, the IP-10 level exhibited transient increase at 21 and 90 days; while the levels of MIP-1α and IL-8 decreased remarkably throughout the whole stress period (21, 90 and 150 days), but jumped up afterwards. Finally the MDC (CCL22) level showed moderate decrease, and did not return to the baseline at 300 days’ time point (Figure 2B, Supplementary Figure 6). Among growth factor family members, EGF and VEGF showed transient increase while TGF- exhibited moderate decrease during the stress period (Figure 2C, Supplementary Figure 7).

### Chronic social restriction alters the immune cell populations

Cytokines binds to cell surface receptors and modulate immune functions. The changes in cytokine might either cause the dynamics in immune cell populations or reflect the functions of secreting immune cells. Therefore we further investigated the changes in immune cell populations in the serum via fluorescence sorted flow cytometry to examine CD3^+^ total T cells, CD8^+^ T suppressor cells, CD4^+^ T helper cells, CD45^+^CD19^+^ B cells, and CD16^+^CD56^+^ NK cells. We found that with minor changes in total T cells and T suppressor cells, the T helper cells increased at different time points of the stress, which did not return to the baseline at 300 days (Figure 2D, Supplementary Figures 8, 9). On the other hand, at 300 days the percentage of B cells actually decreased; whether this is due to the decrease of B cells in numbers, or increased percentage of other lymph cells is yet unknown.

### Cytokine changes act as potential diagnostic biomarkers of stress-relevant diseases

We wonder if these observed changes of serum molecules could potentially act as valid biomarkers for diagnosis of stress-related disorders as the co-morbidities. The receiver operating characteristic (ROC) curve revealed that the changes in some chemokines could act as the diagnostic biomarkers. The sensitivity and specificity for MIP-1α were 90.0% and 80.0% (*p* < 0.001), and for IL-8 were 93.3% and 70.0% (*p* = 0.001), respectively (Figure 2E, Supplementary Table 1), suggesting for their validity as potential biomarkers for stress-related disorders.

Given the heterogeneity of psychological and physiological responses to stress in outbreed species, we asked if MIP-1α can act as an indicator of vulnerability to social restriction stress across different individuals. Correlation analyses confirmed the association between the decreased MIP-1α levels and the increased hostility (determined by the psychological scales) at day 90 (peak of the cortisol response) (Figure 2F, Supplementary Figures 10, 11, 12 and 13), as well as other day points. Therefore the decrease of serum MIP-1α might be used as a marker of personal responsivity in stress-related disorders. However, IL-8 showed no correlation to hostility differences among different individuals; both MIP-1α and IL-8 were not responsible for the anxiety level among different individuals.

## DISCUSSION

The social status such as the hierarchy leads to adrenocortical, cardiovascular, reproductive, immunological, and neurobiological consequences in primates [7]; in addition, social restriction leads to immune change in rodents, dogs and non-human primates [8–10]. Here we found the changes in both cortisol secretion and circulating cytokines after social restriction stress in healthy human subjects. On one hand, the glucocorticoid (cortisol for human) signaling can suppress the inflammatory reactions [11], and regulate the Th1/Th2 balance [12]. While with prolonged stress (chronic phase), the glucocorticoid resistance leads to reduced negative feedback effect of cortisol on inflammation [13, 14]. In addition, the glucocorticoids can be pro-inflammatory at both peripheral and central sites [15, 16]. The detailed interactions between cortisol secretion and cytokine changes are to be dissected in future studies.

Previous studies on human immune system changes after chronic stress (e.g. job stress, low socioeconomic status, childhood adversities, caregiver stress and loneliness) mainly focused on certain cytokines, especially IL-6 due to its enrichment in the serum [3]. The present study as well found the serum increase of IL-6 in the prolonged phase of chronic stress of social restriction. In addition, we have identified an arsenal of cytokines that underwent dynamic changes within the stress period. This is to our knowledge, the first systemic investigation of human cytokines in chronic psychosocial stress stimuli. In addition, we were the first to find that MIP-1α is an important cytokine related to chronic stress and maybe served as a potential biomarker for stress related disorder. These observations is valuable and need to be further investigated.

In summary, the present study demonstrated that chronic social restriction stress triggered dynamic adaptations in human immune system, reflected in the concentration of different cytokines and immune cells, some of which did not return to baseline even after 5 months of the stressor removal. This reveals that the nervous system is tightly linked to the immune system function, potentially via the endocrine mechanisms. These findings expanded our understanding about the decreased immune functions in people working under chronic stress, and opened up the possibility to identify the personal vulnerability with certain biomarkers. To date, the complete serum proteome and the biochemical spectrum remain to be further investigated to search for other biomarker candidates that may contribute to the immune system changes upon different chronic stressors.

## MATERIALS AND METHODS

### Human subjects

10 male healthy volunteers (aged 18-30 years old) following strict physical examinations selections were recruited for present study. All subjects have no physical disease, no alcohol abuse/drug use history, nor psychological/mental disorders (measured by SCL-90, DMEM IV scales). Upon admission, all subjects demonstrated normal hostility and anxiety upon psychological scale measurements. The volunteers were paid with a monthly salary that was equal to the average income in the local city (Hangzhou) during the whole experimental period. The volunteers do not know each other in prior to the experiment.

All the volunteers read the complete description of the study, and signed the written informed consents. All procedures were approved by the Ethics Committee for human research of the Zhejiang Sci-tech University, and were in accordance to the approved guidelines of human medical research.

### Grouped social restriction

The subjects underwent 150 days social restriction period, which was divided into 6 stages (22 days continuously with 3 days off to comply the ethics regulations). During the social restriction period, the 10 volunteers stayed in two connected rooms of 50 square meters in total (Figure 1A), and have no access to communication tools or information updates, such as print media, mobile phone and internet. The TV and computer in the room are used for offline-games or DVDs only. Infrared cameras were used to monitor their safety through the whole experiment.

During the grouped social restriction, no hierarchy was built among the volunteers and no conflict within the group occurred. Within the restriction period, the volunteers mainly employed computer and TV for entertainments (offline). There were no significant differences in amount of physical exercise/TV watching/hour of playing/sleep length across the individuals; during the off period, the volunteers were let free but not for binge activities (e.g. drinking, smoking).

### Blood samples collection and detection

The venous blood samples were collected in early morning at 8 am after overnight fastening (following normal sleep at night). The blood cells were processed for cell flow cytometry (FACSCalibur, Becton–Dickinson) with the BD Multitest™ IMK kit. The serum samples were subjected for series of biochemical examinations for cytokines using the MILLIPLEX MAP Human Cytokine/Chemokine Magnetic Bead Panel - Premixed 39 Plex kit (Millipore, Billerica, Massachusetts, USA) and performed on Luminex 200 system (Luminex, Austin, TX, USA). The serum samples were also detected for cortisol and IL-6 using the Cortisol Assay (R&D Systems, Minneapolis, MN, USA) and the Human IL-6 Quantikine High Sensitivity Immunoassay kit (R & D Systems, Minneapolis, Minnesota, USA), respectively.

### Psychological measurements

The subjects were periodically examined for psychological status with different scales on the day of the blood sample collection. The employed scales included modified State Hostility Scale (SHS) and Subjective Emotion Scale (SES). The modified SHS was with high reliability and validity for Chinese population in previous trials (Data not shown).

### Data processing

The data at day 0 was considered as the baseline control for following experiments. There were no specific event/activity reported in the previous week to the start point of the experiment, and all volunteers were in normal physiological and psychological conditions (measured by psychological scales).

In order to compare the relative changes across different cytokines, the absolute concentration of cytokines were normalized to day 0 (100%) in each individual.

The paired *t*-test was used to compare the changes of different parameters in different time points with baseline. With the data at day 30, 90 and 150 with prolonged stress was served as treatment group, and the data at baseline and the day 300 when the stress had been removed was served as control group, Receiver operating characteristic (ROC) curves were performed to determine diagnostic accuracy of different cytokines for chronic stress. Differences were considered significant at a 2-tailed *p* < 0.05. SPSS (Statistical Package for the Social Sciences) v17.0 was used for all statistical analyses.

## SUPPLEMENTARY MATERIALS FIGURES AND TABLE


