# Supplementary material for: Chronic grouped social restriction triggers long-lasting immune system adaptations
Source: Oncotarget. 2017 Apr 5;8(20):33652–7. doi: 10.18632/oncotarget.16856 (PMC5464898; doi:10.18632/oncotarget.16856)
Supplement: Supplementary file 1 [file oncotarget-08-33652-s001.pdf]

Chronic grouped social restriction triggers long-lasting immune system adaptations

Supplementary Materials

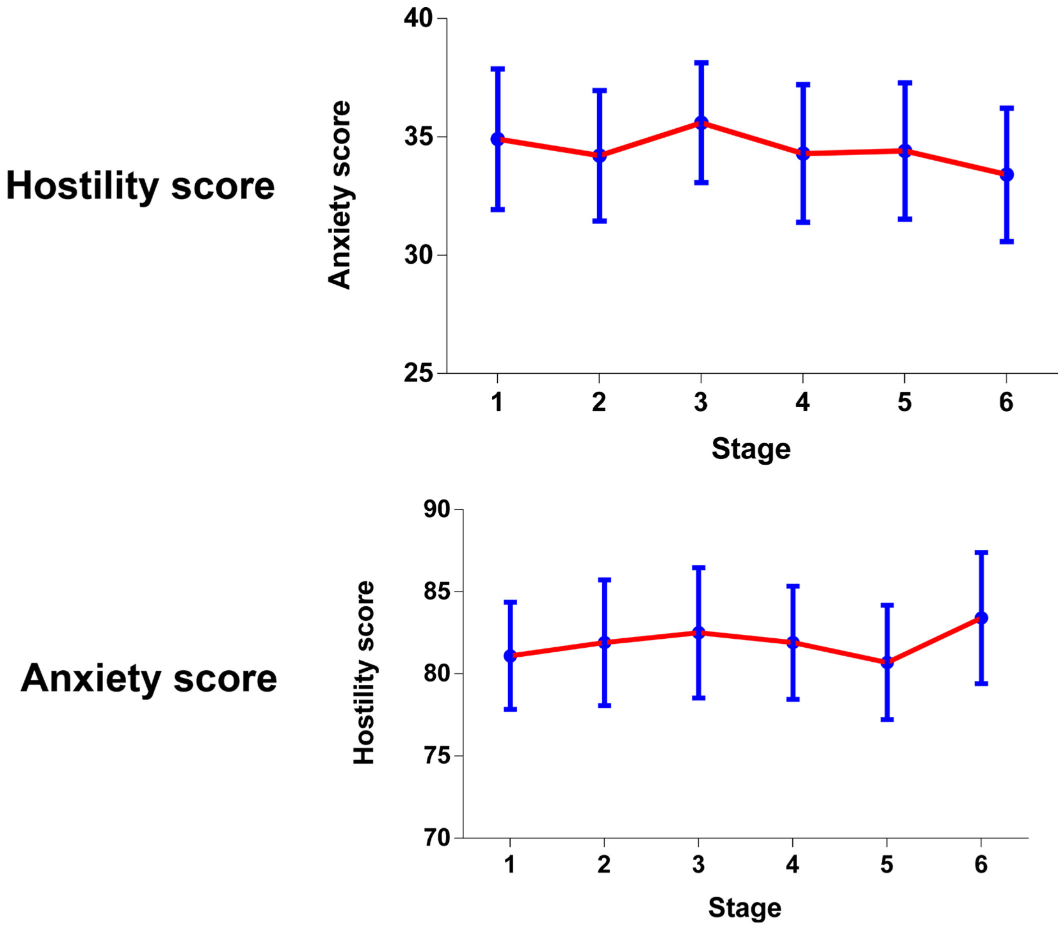

Supplementary Figure 1: The Hostility score (SHS scale) and anxiety score of 10 subjects at different stages of the social restriction period (every 25 days for a total of 150 days).

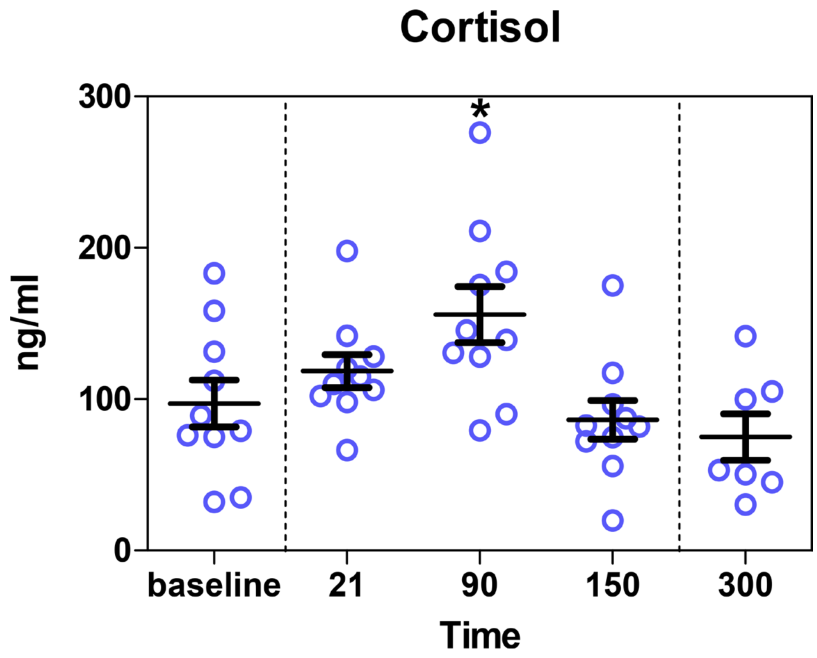

Supplementary Figure 2: The serum cortisol concentration at different time points (*n* = 10).

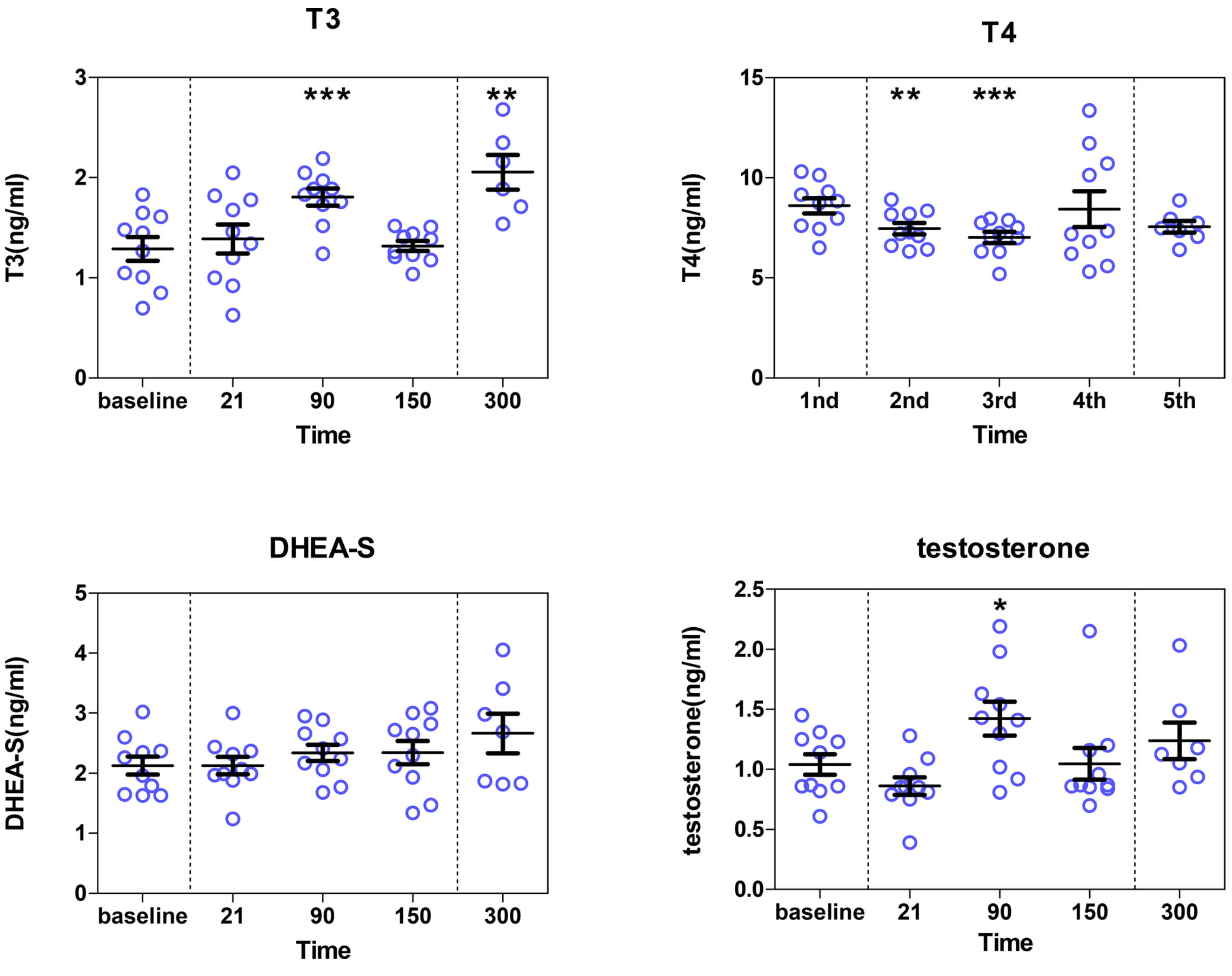

Supplementary Figure 3: The serum T3, T4, DHEA-S and testosterone concentration at different time points (*n* = 10).

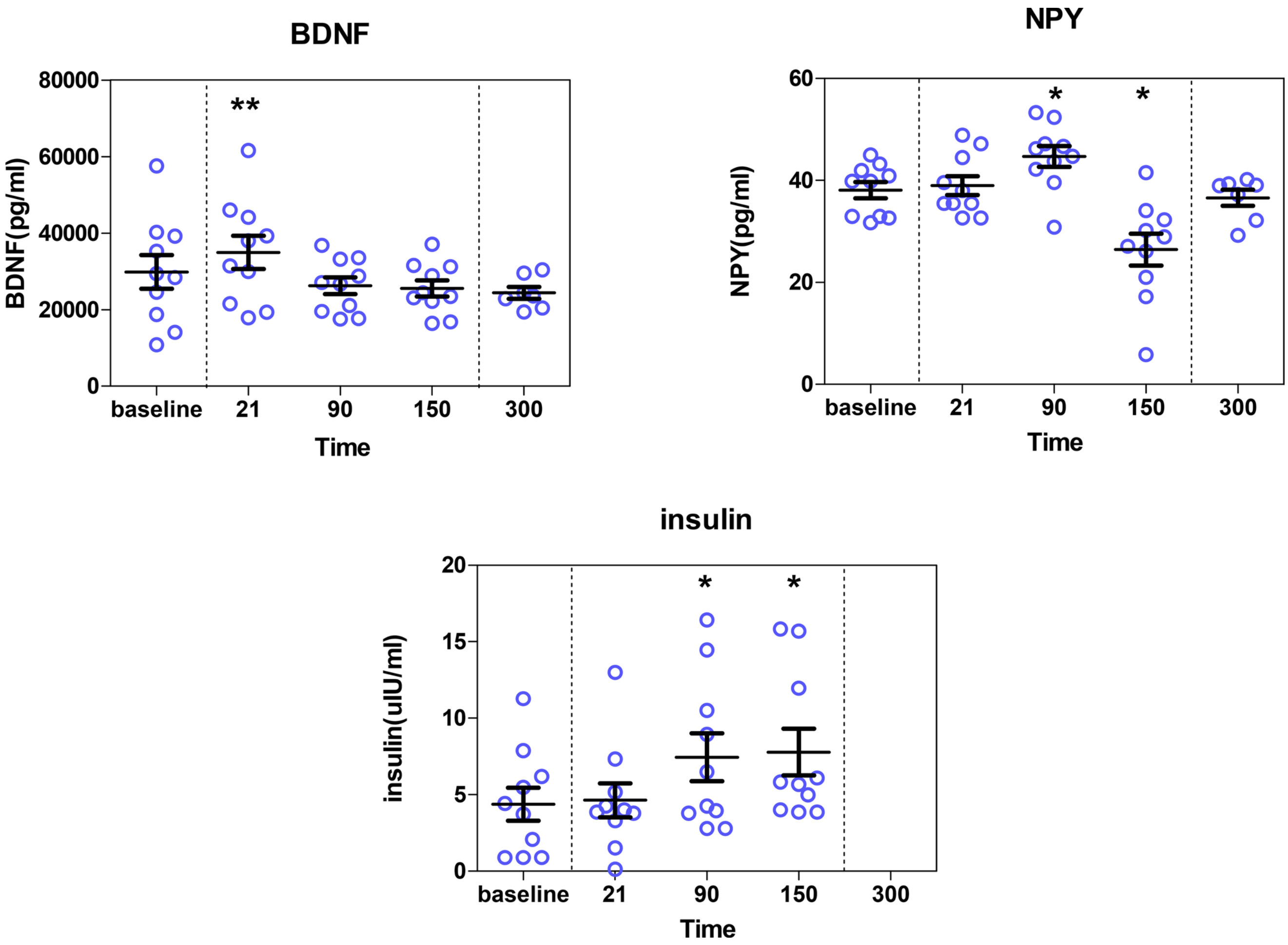

Supplementary Figure 4: The serum BDNF, NPY, and insulin concentration at different time points (*n* = 10).

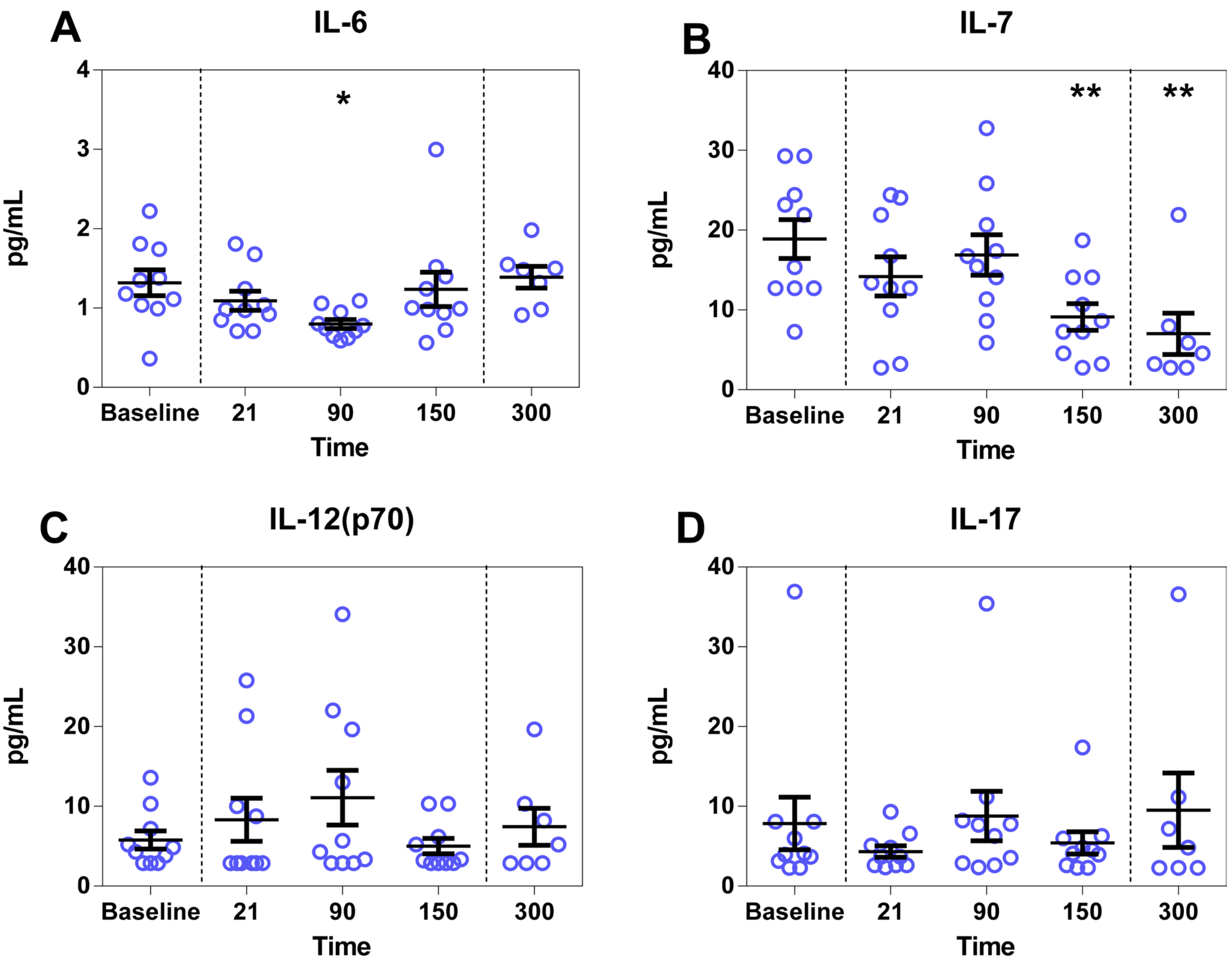

Supplementary Figure 5: The serum cytokine IL6,7,12 and 17 concentration at different time points (*n* = 10).

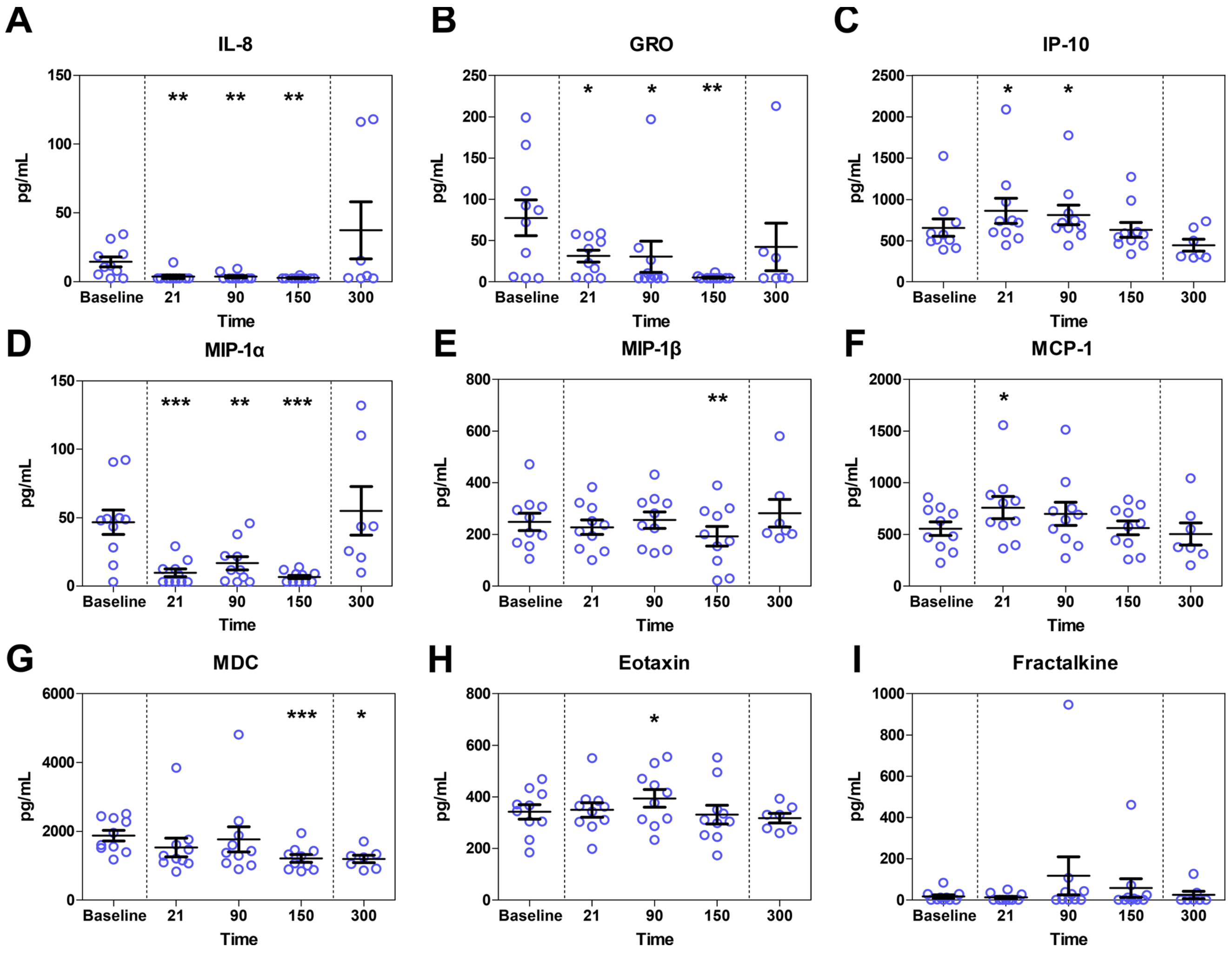

Supplementary Figure 6: The serum cytokine IL-8, GRO, IP-10, MIP-1alpha, MIP1-beta, MCP-1, MDC, Eotaxin and Fractalkine concentration at different time points (*n* = 10).

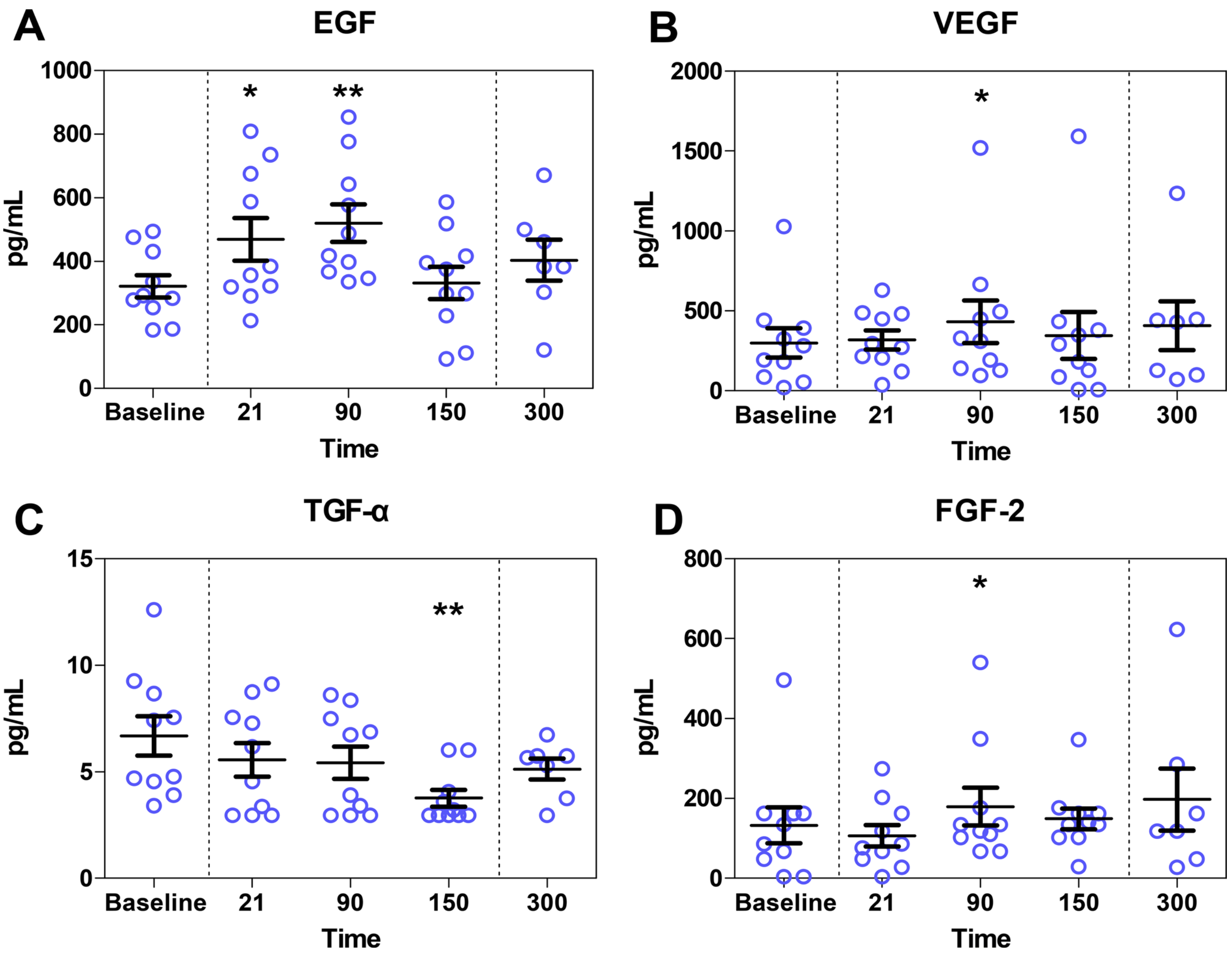

Supplementary Figure 7: The serum EGF, VEGF, TGF-alpha and FGF-2 concentration at different time points (n = 10).

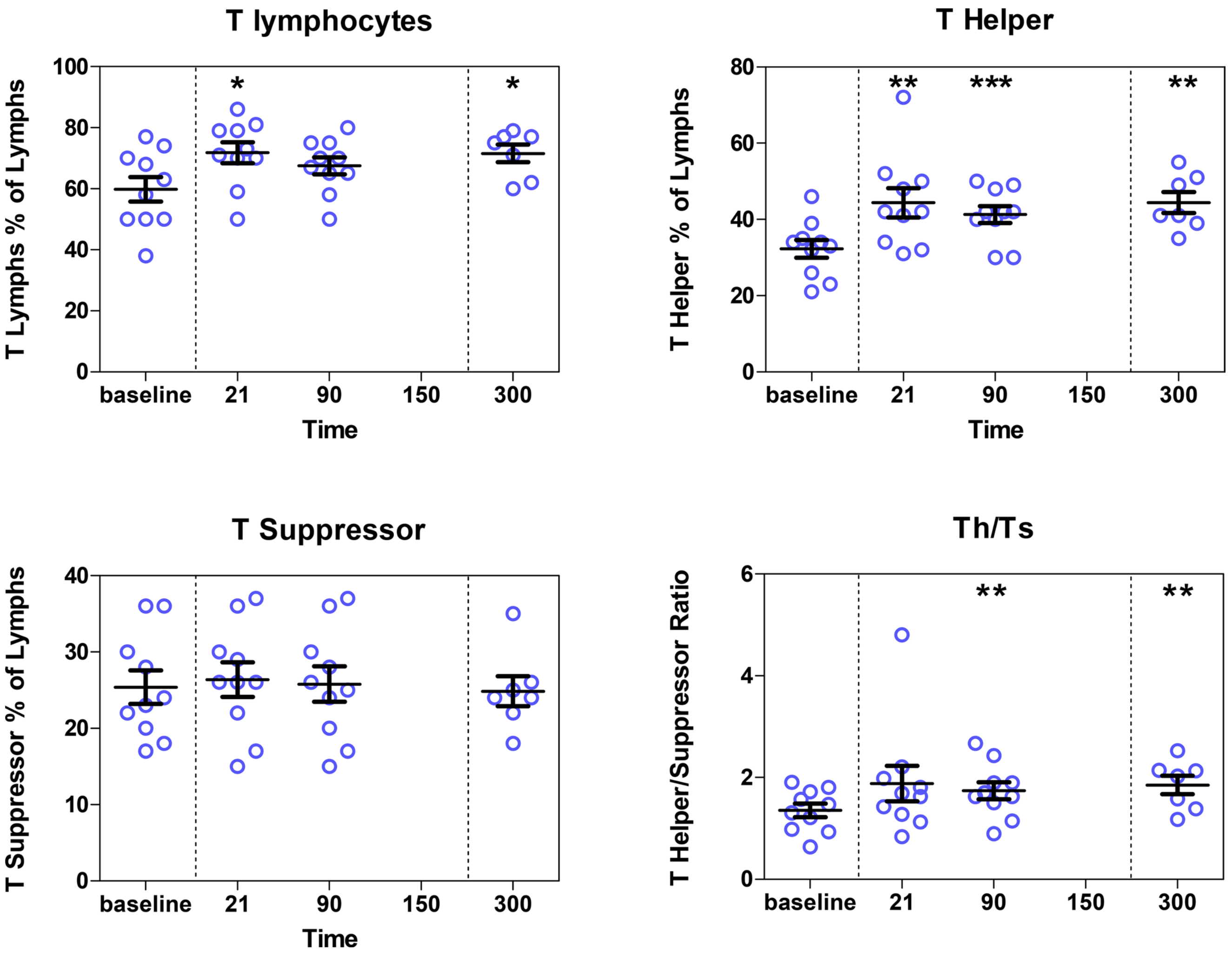

Supplementary Figure 8: The lymph cell percentage at different time points (*n* = 10).

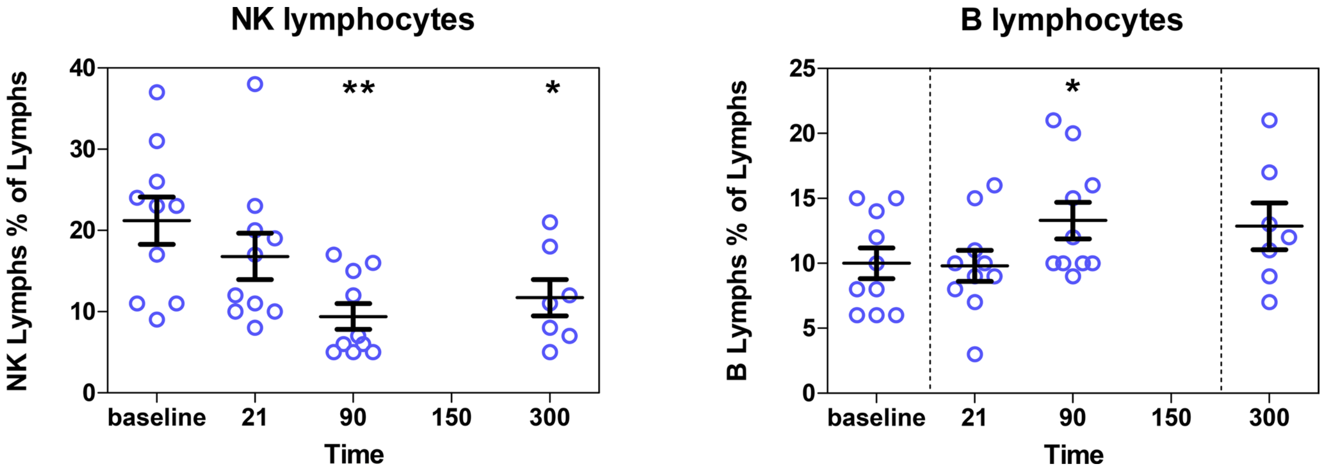

Supplementary Figure 9: The lymph cell percentage at different time points (*n* = 10).

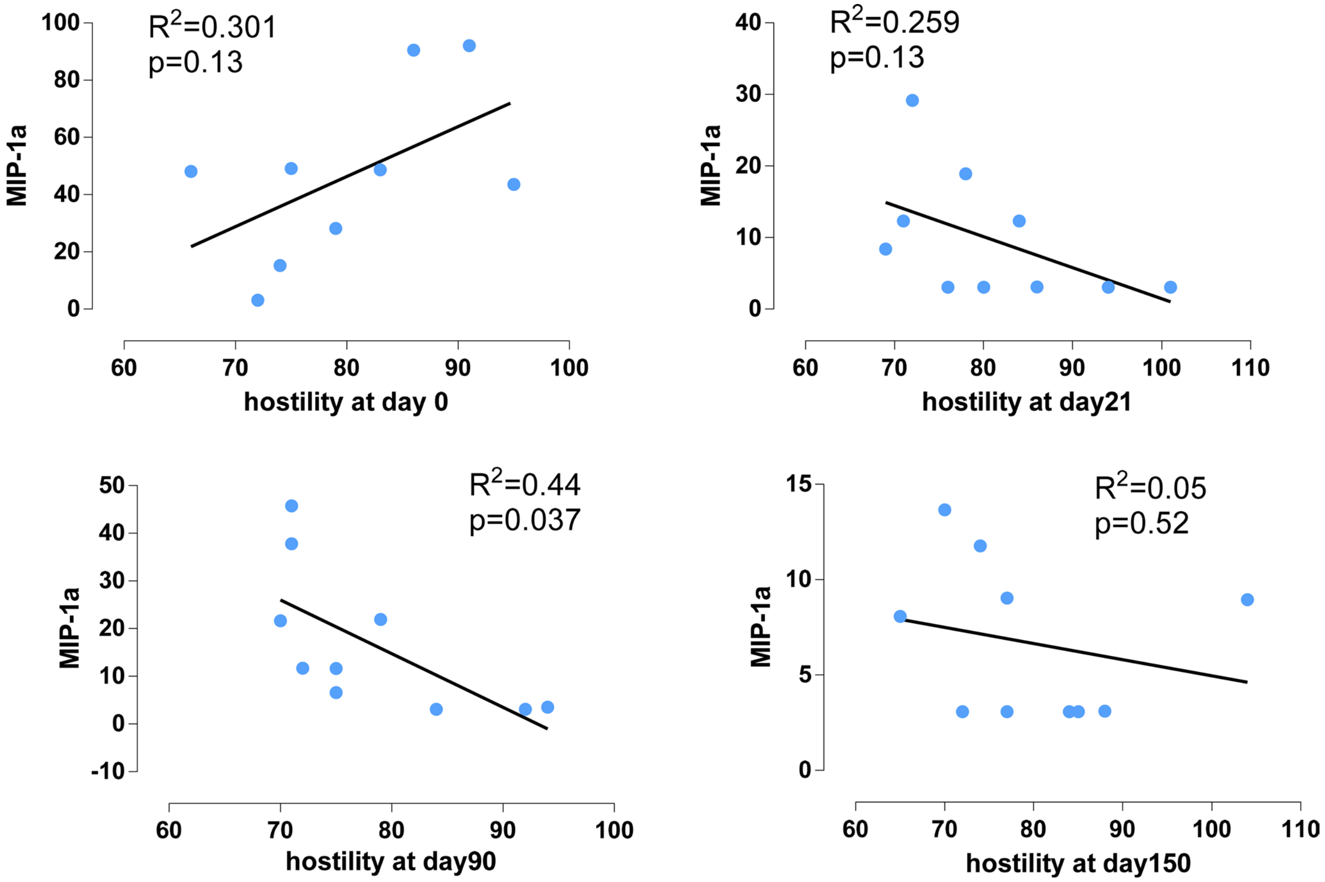

Supplementary Figure 10: The individual difference in hostility is correlated to MIP-1alpha concentration (*n* = 10).

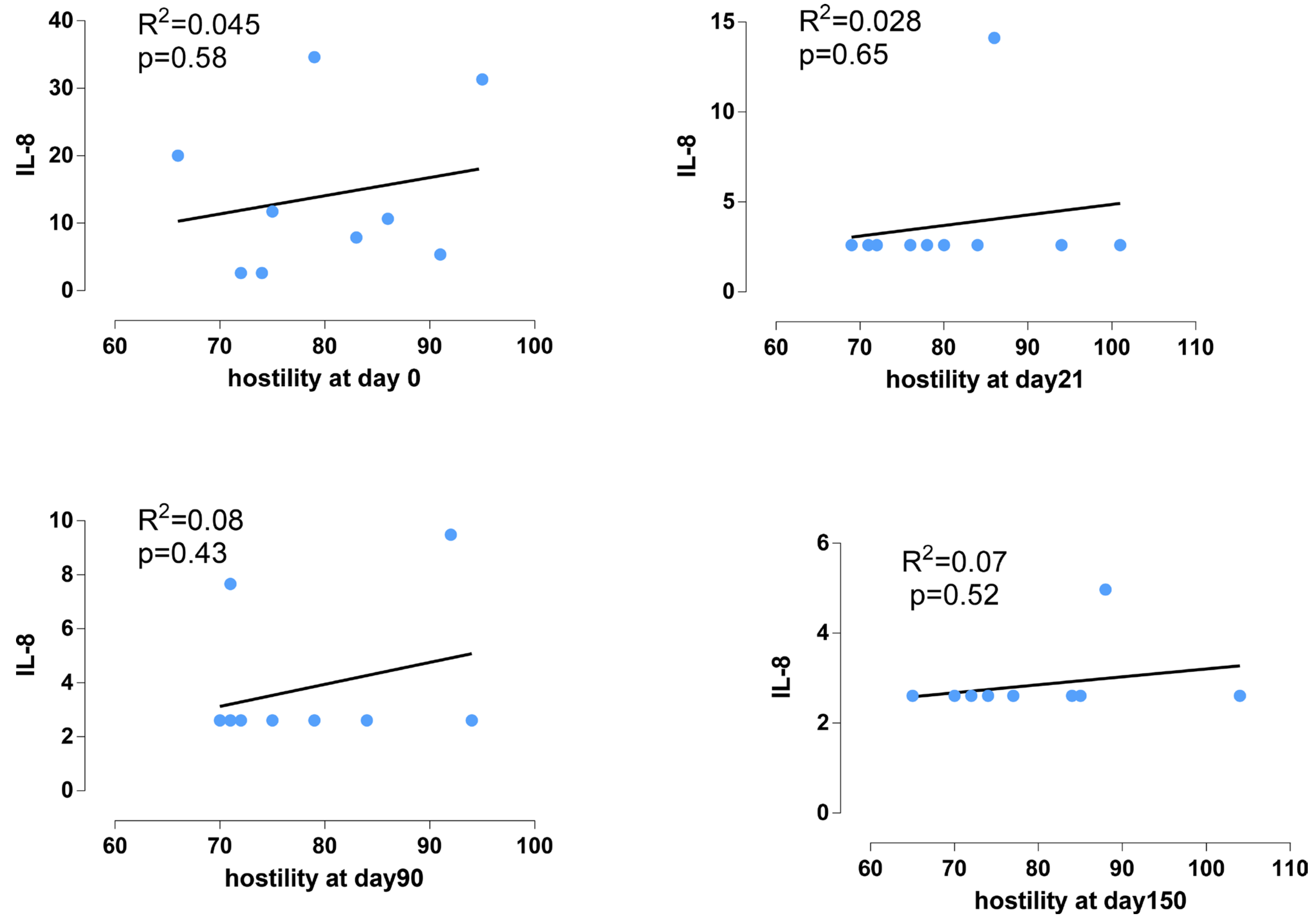

Supplementary Figure 11: The individual difference in hostility is not correlated to IL-8 concentration (*n* = 10).

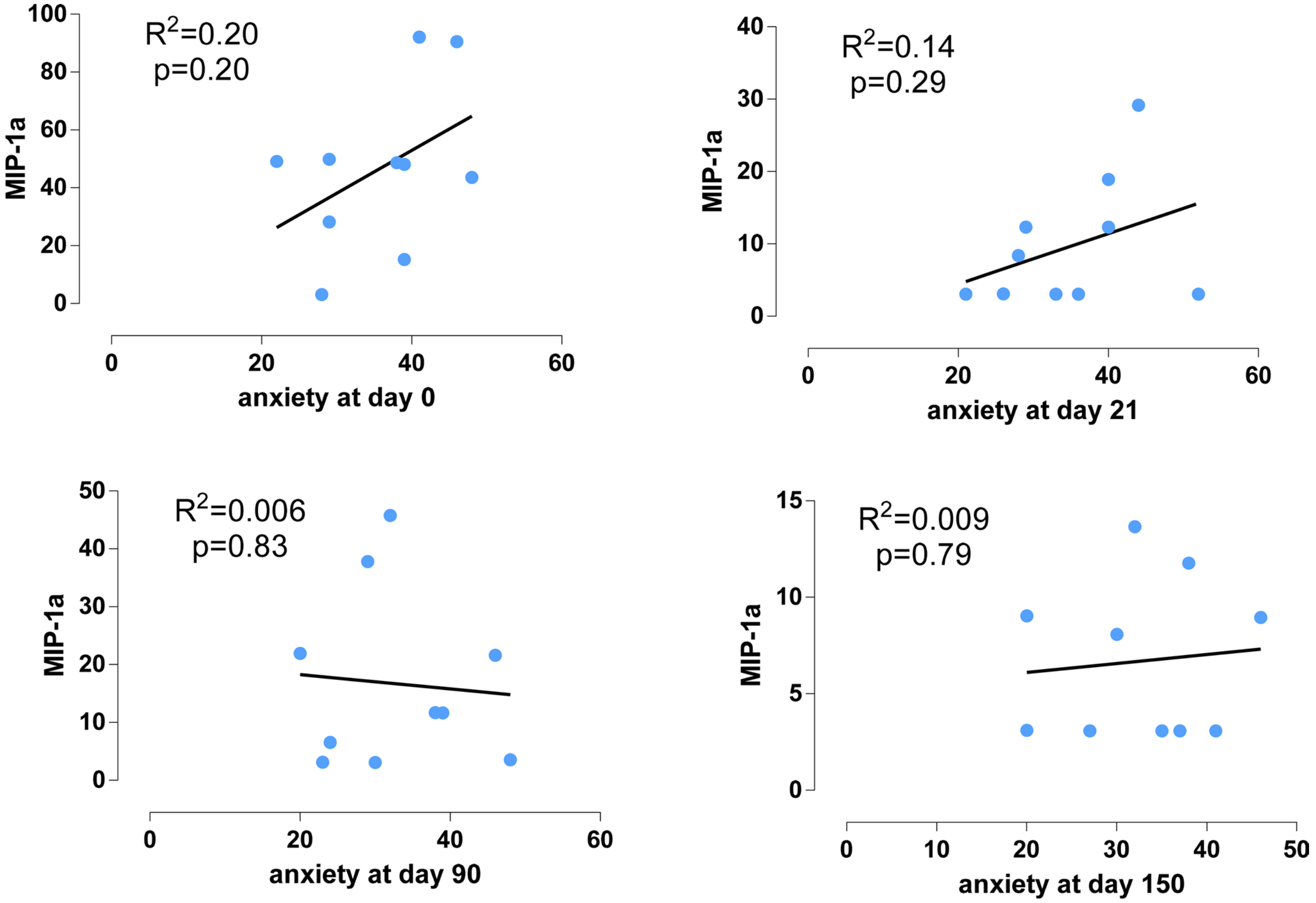

Supplementary Figure 12: The individual difference in anxiety is not correlated to MIP-1alpha concentration (*n* = 10).

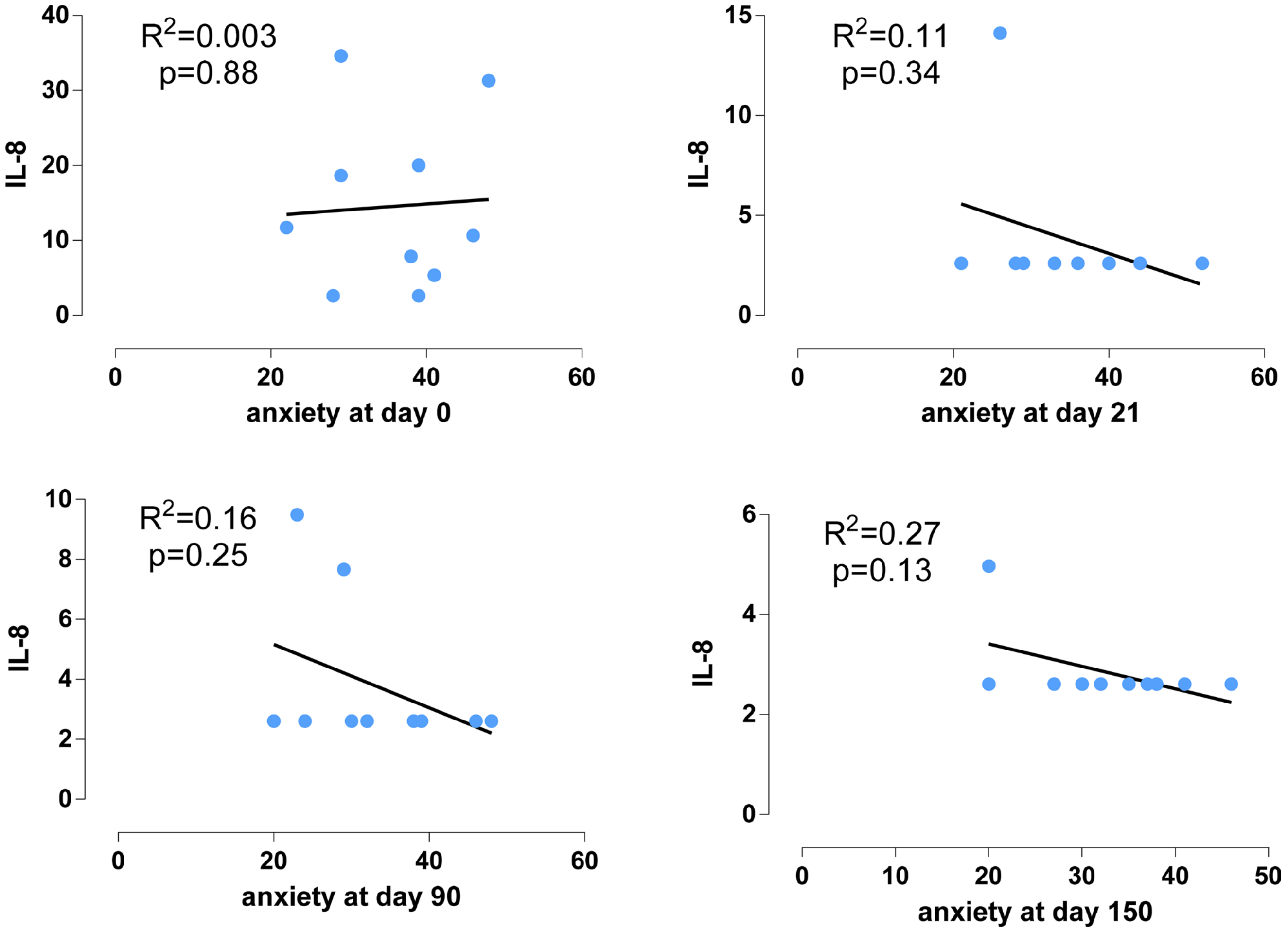

Supplementary Figure 13: The individual difference in anxiety is not correlated to IL-8 concentration (*n* = 10).

Supplementary Table 1: Optimal cut-off value, area under the ROC-curve (Area), and diagnostic sensitivity and specificity of statistically significant biochemical markers

Name	Area	Sig	Cut-off value	Sensitivity	Specificity%
**MIP-1α**	0.882	< 0.001	25.06	90.0	80.0
**IL-8**	0.863	0.001	7.765	93.3	70.0
**MDC**	0.780	0.009	1492.5	73.3	80.0
**GRO**	0.763	0.014	30.725	76.7	70.0
**IL-6**	0.735	0.028	0.985	60.0	90.0
**TGF-α**	0.732	0.030	4.305	56.7	80.0
**EGF**	0.705	0.055	340.5	66.7	70.0

The sensitivity and specificity of potential biomarkers reflected by ROC-curve
